# Dendritic cell vaccine of gliomas: challenges from bench to bed

**DOI:** 10.3389/fimmu.2023.1259562

**Published:** 2023-09-14

**Authors:** Ye Zheng, Xiaoyu Ma, Shouchang Feng, Hongtao Zhu, Xin Chen, Xingjiang Yu, Kai Shu, Suojun Zhang

**Affiliations:** ^1^ Department of Neurosurgery, Tongji Hospital, Tongji Medical College, Huazhong University of Science and Technology, Wuhan, China; ^2^ Department of Oncology, Tongji Hospital, Tongji Medical College, Huazhong University of Science and Technology, Wuhan, China; ^3^ Department of Histology and Embryology, School of Basic Medicine, Tongji Medical College, Huazhong University of Science and Technology, Wuhan, China

**Keywords:** dendritic cell vaccine, glioma microenvironment, immunotherapy, glioma, dendritic cell

## Abstract

Gliomas account for the majority of brain malignant tumors. As the most malignant subtype of glioma, glioblastoma (GBM) is barely effectively treated by traditional therapies (surgery combined with radiochemotherapy), resulting in poor prognosis. Meanwhile, due to its “cold tumor” phenotype, GBM fails to respond to multiple immunotherapies. As its capacity to prime T cell response, dendritic cells (DCs) are essential to anti-tumor immunity. In recent years, as a therapeutic method, dendritic cell vaccine (DCV) has been immensely developed. However, there have long been obstacles that limit the use of DCV yet to be tackled. As is shown in the following review, the role of DCs in anti-tumor immunity and the inhibitory effects of tumor microenvironment (TME) on DCs are described, the previous clinical trials of DCV in the treatment of GBM are summarized, and the challenges and possible development directions of DCV are analyzed.

## 1 Introduction

Diffuse glioma is diagnosed in approximately 100,000 people worldwide each year. Although it accounts for a small proportion (~1%) of all newly diagnosed cancers, diffuse glioma is related to high morbidity and mortality (1). According to the fifth edition of the World Health Organization (WHO) Classification of Tumors of the Central Nervous System, adult diffuse gliomas consist of three types: astrocytoma, IDH mutant (IDHmut); oligodendroglioma, IDHmut and 1p/19q co-deletion; glioblastoma (GBM), IDH wild type (IDHwt) (2). Glioblastoma is the most fatal subtype of glioma, accounting for 70 to 75% of all diffuse gliomas diagnosed, with a median overall survival range from 14 to 17 months (1).

Currently, first-line therapy for GBM typically consists of maximally safe resection followed by adjuvant temozolomide chemotherapy, concurrent fractionated radiotherapy, and maintenance temozolomide chemotherapy (3). This multimodality approach significantly improves survival. However, the prognosis is still quite poor and the relapse of GBM is common, with a median survival of only 6.2 months after relapse. To date, the main treatment options for recurrent GBM, including tumor-treating field (TTF) therapy, lomustine, carmustine, and the antiangiogenic agent bevacizumab (4, 5), are barely effective. Therefore, there is an urgent need to find more effective treatments for GBM.

**Figure 1:**
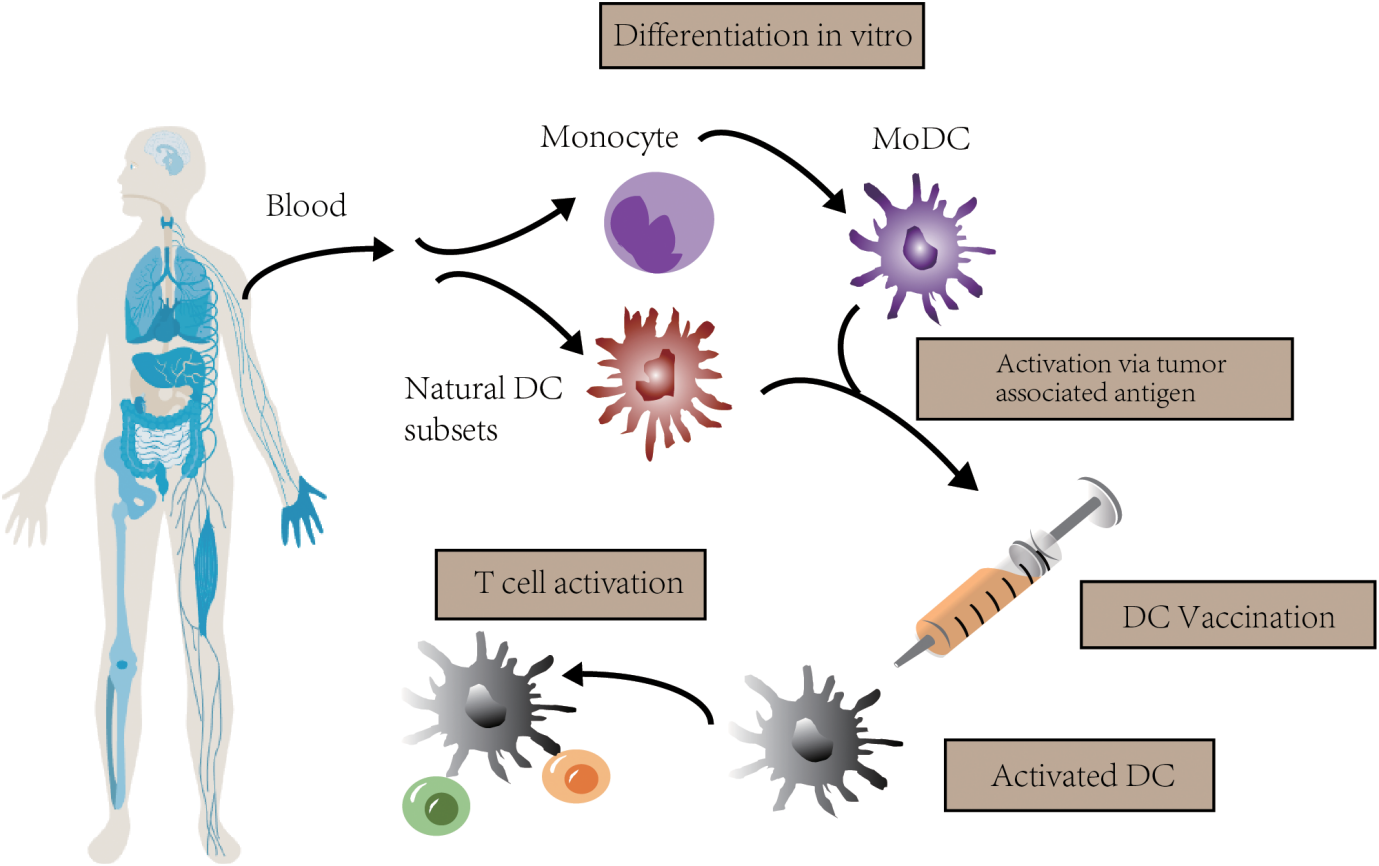
The concept of DC vaccination. In clinical trials, autologous monocytes are commonly used as the source of DC vaccines with differentiation and maturation *in vitro*. After loaded with tumor associated antigen, DC vaccines are infused to activate T cell response.

Dendritic cells (DCs) are a kind of professional antigen-presenting cells (APCs) that are essential for the T cell response. They present extracellular antigens to CD4^+^ T helper (TH) cells via major histocompatibility complex (MHC) class II molecules, and present intracellular antigens to CD8^+^ T cells via MHC class I molecules. This so-called “cross-presentation” phenomenon, takes an important part in antitumor immune responses (6). DC vaccine is a kind of immunotherapy based on the effect of DC. The blueprint is that patients are administrated with DCs activated by tumor-associated antigens (TAAs), inducing an antitumor T cell response. This response eliminates tumor cells selectively and prevents tumor relapse because of immunologic memory (7, 8) (**Figure 1**).

In recent decades, many advances have been achieved in the use of DC vaccines (DCV) in anti-tumor therapy. Since DCV made its debut in a B cell lymphoma clinical trial in 1996 (9), a large number of preclinical and clinical trials have been conducted using DCV for various tumors (10, 11). However, to date, only one tumor-targeted DCV therapy, sipuleucel-T, has Food and Drug Administration (FDA) approval to treat metastatic castration-resistant prostate cancer (12). The number of clinical trials using DCV has decreased significantly in recent years. This partly results from the rapid development of alternative immunotherapeutic methods, such as immune checkpoint inhibitors (ICIs) (13–15), and partly results from the disappointing clinical performance of DCV.

Nevertheless, due to the unique immune microenvironment of GBM, many immunotherapies that have successfully treated other tumors do not work in GBM (16, 17). As a result, there has been renewed interest in using DCV for treating GBM, particularly when it is combined with conventional therapies (e.g., chemotherapy, radiation) or other immunotherapies (e.g., ICIs) (18–20). Clinical trial results have varied widely, with clinical responses ranging from minimal to significant. Overall, although there are some promising results, conclusive evidence is still lacking. In this review, we analyze the role DC plays in antitumor immunity and the immunosuppressive effect of the tumor microenvironment (TME) on DC, summarize clinical trials that have used DCV for treating GBM in recent years, and propose the challenges and possible development directions of DCV.

## 2 DCs in anti-tumor immunology

At present, our understanding of DC subsets and functions comes mainly from murine models, while recently the number of studies aimed at assessing the biological properties of human DCs has significantly increased. According to the differences in development, phenotype, and function (See **Table 1**), DCs can be classified into several subtypes: classical, or conventional DCs (cDCs), plasmacytoid DCs (pDCs), and monocyte-derived DCs (MoDCs), etc (7). cDCs, which consist of two major subsets: cDC1s and cDC2s, develop from common DC precursor cells (CDPs) in the bone marrow (21). In humans, cDC1s can be recognized by highly specific cell surface markers such as CD141, XCR1, CLEC9A, and DEC205 (7, 21–23). cDC2s are more heterogeneous in cell surface markers and can be further classified into CD5^+^ cDC2s (DC2s) and CD5^-^ cDC2s (DC3s) based on the presence or absence of CD5 expression (22, 24). It remains unknown whether DC3s are derived from CDPs (22). In addition, some studies have suggested the existence of other subsets of cDC2, such as DC4 (CD1C^-^ CD141^-^) and DC5 (AXL^+^ SIGLEC6^+^), whose classification and function require further study (24). Although still controversial, current studies suggest that pDCs arise from both CDPs and lymphoid progenitors and that pDCs from different sources have different functions. During acute or chronic viral infection, mature pDC subsets of both different origins can secrete type I interferon, but only bone marrow-derived pDCs can process and present antigens (25, 26). Furthermore, pDCs are involved in the progression of autoimmune diseases (27), and the high frequency of pDCs in tumors is highly correlated with poor prognosis (28). In contrast to cDCs and pDCs, MoDCs originate in monocytes. Under the context of inflammation, monocytes in the blood are recruited through CC chemokine 2 (CCR2)-dependent pathways and differentiate into MoDCs in peripheral tissues (23). In response to inflammation, MoDCs allow CD4^+^ T cells’ differentiation into TH1, TH2, or IL-17-producing TH cell (TH17) phenotypes, depending on the context (29). Some investigators have suggested that CD16^+^ non-classical monocytes are also a type of DC, particularly those expressing carbohydrate-modified P-selectin glycoprotein ligand 1 (slanDCs) (23), which have potent pro-inflammatory properties.

**Table 1:** DC subsets in human.

DC subset	Origin	Main surface markers	Main functions
cDC1s	CDPs	CD11c^low^, HLA-DR^+^, CD141^+^, XCR1^+^, CLEC9A^+^, DEC205^+^	Recruit CD8+ T cells to tumors, induce TH1 responses by IFN-λ production, mediate TH type 1 (TH1) polarization of CD4+ T cells.
cDC2s	CDPs	CD11c^+^, HLA-DR^+^, CD1c^+^, CD11b^+^, CD172a^+^, CD1a^+^, CD14, CD5	Secrete proinflammatory and anti-inflammatory cytokines, including IL-12. Required for initiating antitumor CD4+ T cell responses.
pDCs	CDPs/lymphoid progenitors	CD11c^-^, HLA-DR^low^, CD123^+^, CD303^+^, CD304^+^, CCR2^+^, CXCR3^+^	Have the strongest type I IFN responses, the major producers of IFN-α, related to poor prognosis in various cancers. Only bone marrow-derived pDCs can process and present antigens, involved in the progression of autoimmune diseases.
MoDCs	Monocytes	CD11c^+^, HLA-DR^+^, CD1c^+^, CD11b^+^, CD14^+^, CD64^+^, CD206^+^, CD209^+^, CD172a^+^, CD1a^+^, CCR2^+^	Depending on the context, allow CD4+ T cells` differentiation into TH1, TH2, or IL-17-producing TH cell (TH17) phenotypes.

DCs remain immature when pathophysiological stimuli are absent, and are crucial to immune surveillance (30, 31). Immature DCs (iDCs) are indispensable for maintaining tolerance to peripheral autoantigens (32). They can eliminate autoreactive T cells (33), and facilitate the expansion and differentiation of regulatory T cells (Tregs) (34). iDCs mature when they encounter microbial stimuli or endogenous stimuli associated with inflammation (35). Reduced phagocytic activity, increased expression of costimulatory ligands and MHC class I/II molecules on the cell surface, expression of chemokine receptors involved in lymph node homing and retention, and increased secretion of chemokines and proinflammatory cytokines are the main differences between mature DCs (mDCs) and iDCs (10).

cDC1s are of great importance in anti-tumor immunity and are the mere type of APC that effectively primes tumor-specific CD8^+^ T cells (36). In both murine and humans, cDC1s are crucial for the recruitment of CD8^+^ T cells to tumors (37). cDC1 is a major producer of IFN-λ, which induces TH1 responses (38, 39). cDC1s can also mediate TH type 1 (TH1) polarization of CD4^+^ T cells (40). Thus, the abundance of cDC1 in the tumor microenvironment (TME) has a positive correlation with patient survival (37). On the contrary, the understanding of the functions of cDC2s in antitumor responses is relatively new. cDC2s secrete a variety of cytokines, some of which are anti-inflammatory while some of which are pro-inflammatory, including interleukin-12 (IL-12), which is essential for the expansion and survival of T cells and natural killer (NK) cells (41, 42). cDC2s and MoDCs may also have the ability to cross-present antigens, and cDC2s appear to be required for initiating antitumor CD4^+^ T cell responses (7, 43). In addition, cDC2 and MoDC underlie direct or cross-presentation of TAAs after chemotherapy in some cancers (7, 44, 45). Among all the DC subtypes, pDCs have the strongest type I IFN responses and are the major producers of IFN-α (46). In anti-tumor immunity, type I IFNs are thought to be critical for immunogenic responses to anti-tumor therapies. However, high frequencies of pDCs in tumors are related to poor prognosis in a variety of cancers (47, 48). Persistent IFN-I response may be a key factor in immunodeficiency and treatment resistance, although the mechanism is not yet fully understood (49, 50).

## 3 The glioma microenvironment and DCs

### 3.1 The glioma microenvironment

Gliomas, especially GBM, have a unique TME compared to tumors at other sites. The central nervous system (CNS) used to be regarded as an immunologically privileged site. One of the reasons for this understanding is that the lymphatic drainage system of the brain has not been discovered for a long time. Another reason is the existence of the blood-brain barrier (BBB) (51). Recently, the discovery of the glial-lymphatic pathway has proposed a mechanism for connecting the parenchyma and interstitium with the cerebrospinal fluid space (52); meanwhile, the discovery of functional lymphatic vessels in the meninges confirms the existence of a direct drainage pathway for cerebrospinal fluid that contains solutes and immune cells from the brain to the cervical lymph nodes (53, 54). The brain is protected from pathogenic microorganisms by the BBB, consisting of pericytes, astrocyte processes, vascular endothelial cells, and extracellular matrix. Meanwhile, it makes it harder for drugs and peripheral immune cells to enter the CNS, facilitating tumor invasion and growth (55). However, it has been shown in recent studies that T cells can enter the brain and provide immune surveillance (56, 57), challenging the notion that the BBB is sealed to immune cell entry. Simultaneously, damaging the BBB by GBM itself can also limit the BBB’s ability to function (58). In summary, during inflammation, specific antigens are recognized by microglia, then microglia present them to activated lymphoid cells through the glial-lymphoid pathway, and subsequently more immune cells penetrate the BBB, leading to a more intense inflammatory response and following immune responses. Thus, CNS immunity is not so much “privileged” as it is “unique”.

However, compared to other tumor types, CNS tumors have lower levels of tumor-infiltrating lymphocytes (TILs) and other types of immune effector cells (59). This “cold tumor” phenotype has been related to poor response to immunostimulatory therapies such as ICIs (17, 60). Even when inducing T cells to respond to CNS cancer, the number of antigen-specific TILs remains relatively low, and the TILs present often exhibit a depleted phenotype (18, 61). Upon inflammatory stimulation, brain stromal cells produce high levels of classical immunosuppressive cytokines such as TGFβ. These cytokines neutralize inflammatory factors to maintain homeostasis (62). Glioma cells produce high levels of indoleamine-2,3-dioxygenase (IDO), which, besides promoting Treg accumulation, inhibits T-cell activity by depleting microenvironmental tryptophan (63). Microglia and tumor-infiltrating myeloid cells reduce the arginine level in the tissues by producing high levels of arginase, which further suppresses the proliferation and functions of T cells (64). Additionally, the compromised BBB suppresses glioma patients’ adaptive immune response by upregulating programmed death ligand 1/2 (PD-L1/2) expression to prevent effector T-cell from activation (65).

### 3.2 DCs in the glioma microenvironment

Normally, peripheral circulating DCs reach the vascular-rich compartment through the central lymphatic vessels and are virtually absent in the brain parenchyma (66). However, a recent study found that CD141^+^ cDC1 can infiltrate the region of GBM and present antigens to T cells in deep cervical lymph nodes (dcLNs) (67). Nonetheless, extracranial antigen presentation failed to facilitate tumor eradication in the absence of immunotherapy in a melanoma brain metastasis model (68). This indicates that the presentation of antigens in the periphery is probably not sufficient to induce immunity against brain tumors.

**Figure 2:**
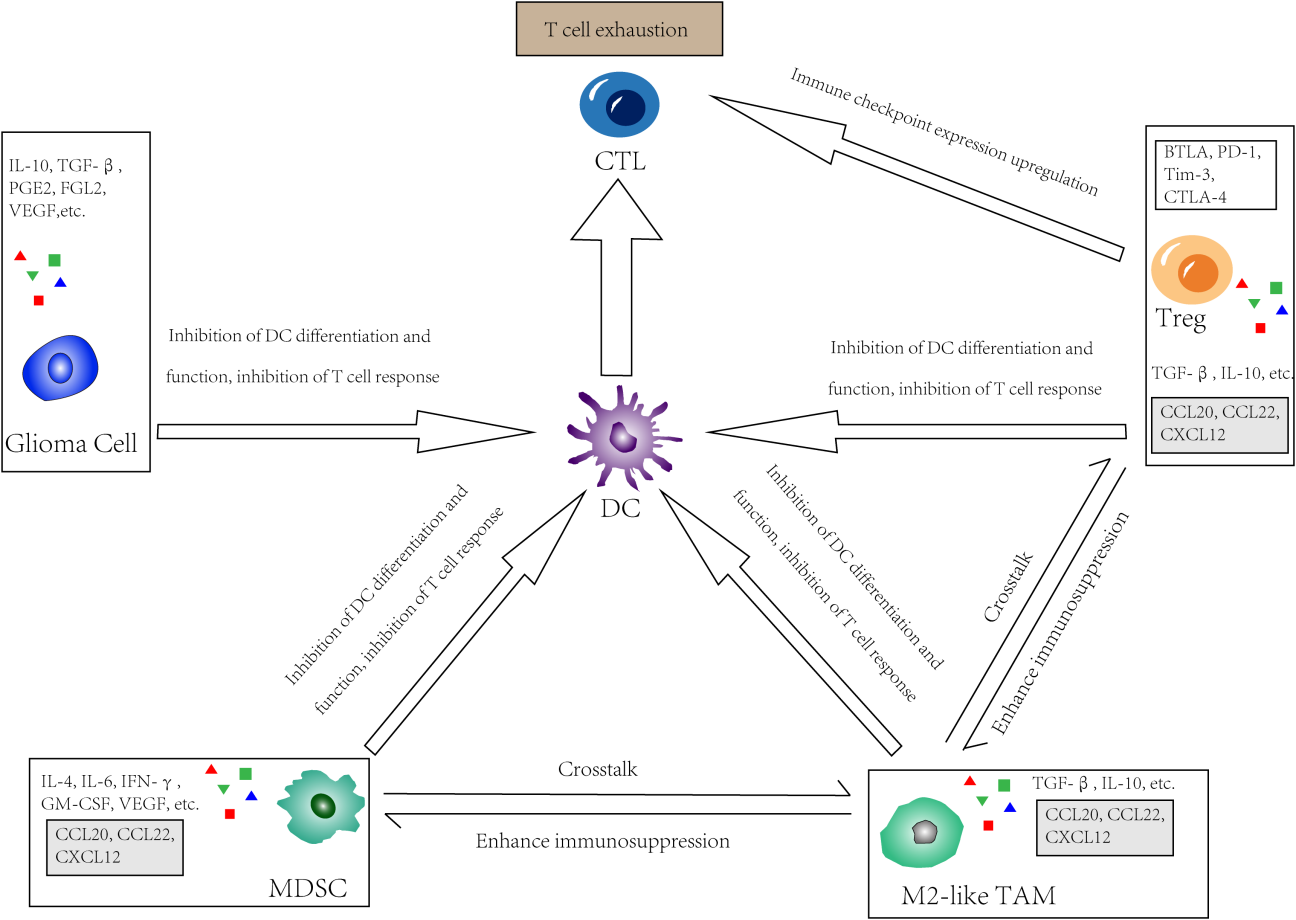
The immunosuppressive glioma microenvironment. In GBM, tumor cells, Tregs, M2-like tumor-associated macrophages (TAMs) as well as myeloid-derived suppressor cells (MDSCs) directly or indirectly inhibit the effect of DC by limit its differentiation and function, or inhibit recruitment, proliferation and function of T cells. Meanwhile, these cells upregulate immune checkpoint expression and interact with receptors on CTLs, thus lead to so called “T cell exhaustion”. There is also a crosstalk in the TME between those cells that secrete chemokines such as CCL20, CCL22, and CXCL12, which further enhancing immunosuppression.

A major barrier to the application of DCs for the treatment of GBM is that DCs must have the capacity to induce anti-tumor immune responses under immunosuppressive conditions. The mechanism of immunosuppression in GBM involves both the glioma cells themselves and the cells in the TME (**Figure 2**).

#### 3.2.1 glioma cells

Glioma cells secrete cytokines like TGFβ, IL-10, VEGF, and CSF-1, which inhibit the differentiation of DCs (69). Glioma cell-produced PGE2 can promote DC-producing IL-10, inhibiting effector T-cell responses (70). Tumor cells release IL-6, inhibiting the CD34^+^ cell differentiation into DCs and promoting the transition of these cells to the monocytic lineage with deficient APC function (71). Some glioma cell products are linked to DC dysfunction, including R-2-hydroxyglutarate (R-2-HG), fibrinogen-like protein 2 (FGL2), Nrf2, etc. In high grade glioma (HGG) patients with IDHmut, reprogramming mediated by the tumor metabolite R-2-HG leads to poor antigen presentation of DCs (66). FGL2 inhibits GM-CSF-induced CD103^+^ DC differentiation through inhibition of NF-κB, STAT1/5, and p38 activation (72). Glioma cells can induce DCs to overexpress Nrf, which inhibits DC maturation and reduces effector T-cell activation (73). Glioma cells can affect DC lipid metabolism, leading to lipid accumulation in DCs and limiting T-cell activation (74). The Warburg effect of glioma cells can lead to lactic acid accumulation, and low pH affects immune cell metabolism and function (75).

#### 3.2.2 Cells in the glioma microenvironment

TME components such as Tregs, myeloid-derived suppressor cells (MDSCs), and tumor-associated macrophages (TAMs) can suppress antitumor immunity by reducing DC responses and causing T cell dysfunction, which is also known as “T cell exhaustion”.

Treg is an immunosuppressive T cell subset that helps to maintain immune tolerance, limits inordinate immune responses, and promotes homeostasis and tissue regeneration. In various solid tumors, the frequency of tumor-infiltrating effector Tregs is high, and the high proportion of Tregs: CD8^+^ T cells is inversely correlated with prognosis (76). Tregs are not detectable in normal brains and are seldom found in low-grade brain tumors. Intriguingly, despite lymphopenia, GBM patients have increased Treg frequencies in TME and blood (77). Treg frequencies vary by glioma subtypes, with higher frequencies in IDHwt than in IDHmut (78). In a murine model of astrocytoma, Tregs accumulate time-dependently after tumor cell implantation. The quantity of Tregs first increases in blood and then in tumor tissue during the asymptomatic phase (79). It can be seen that Tregs are recruited to tumors at an early stage when the number of tumor cells is still low. CD27(TNFRSF) expressed by Tregs can downregulate the expression of CD70 on the membrane of DCs, thereby limiting the activation of CD8^+^ T cells (80). The immune checkpoints BTLA, PD-1, Tim-3, and CTLA-4 expressed on Tregs also limit functions of DCs (65).

MDSCs are a population of immature bone marrow cells that are of high heterogeneity. In the TME, with their strong immunosuppressive activity, they continuously interact with infiltrating T cells, especially cytotoxic T lymphocyte (CTLs), inhibiting their function and thus promoting the growth and progression of tumors (81). MDSCs can be detected in patients with cancer or the setting of chronic inflammation when sustained low-level stimulation of bone marrow cell generation leads to the development of immunosuppressive bone marrow cells (82, 83). First develop in the bone marrow, they then infiltrate and accumulate in solid tumors via factors such as GM-CSF, G-CSF, M-CSF, VEGF, IFN-γ, IL-6, and IL-4, which are secreted by tumor cells or other TME components. In GBM, MDSC is one of the major immunosuppressive components of the TME (84). Recent studies have shown that MDSCs are present in GBM patients’ blood, but not in patients with low-grade gliomas or healthy people (85). MDSCs can prevent CTL entry into tumors and T cell responses to HLA stimulation through ROS- and NO-dependent pathways (86). Like Tregs, MDSCs produce immunosuppressive cytokines such as IL-10 and TGFβ. In addition, MDSCs express immune checkpoint regulatory pathway ligands, such as PD-L1/2 and CD155. These ligands inhibit T-cell responses. When interacting with receptors on T cells, they can even induce T cell apoptosis (87, 88).

TAMs, representing 50% of the total number of living cells in the entire GBM tumor, are the largest immune cell population in the TME of GBM (89). TAMs are a highly heterogeneous cell population, and overall, in both murine and human models, the majority of TAMs in brain tumors seem to originate in circulating monocytes, while approximately 15% of TAMs originate in brain-resident microglia (51, 90). In GBM, however, this heterogeneity depends on the context (e.g., Microglia are relatively abundant in primary GBM, whereas monocyte-derived macrophages predominate in recurrent GBM.) (89). In general, TAMs are thought to promote tumor growth, and the number of TAMs is correlated with tumor grade (91). As is shown in previous *in vitro* studies, macrophages can be classified into two groups: M1 and M2, and the growth-promoting activity of TAM correlates with the M2 macrophage phenotype that is anti-inflammatory. However, TAMs are neither M1-like nor M2-like but exhibit a mixed phenotype (91). In the TME, the majority of TAMs were M2-like cells. Yet, there are also proinflammatory TAMs capable of engulfing tumor cells (92). Immunosuppressive cytokines, such as TGFβ and IL-10, which are expressed by M2-like TAMs, suppress T cell proliferation and function. Meanwhile, they promote extensive crosstalk with Tregs and MDSCs, along with chemokines like CCL20, CCL22, and CXCL12, further enhancing immunosuppression (93, 94).

## 4 Overview of DCV clinical trials

### 4.1 The source of DCs

A large majority of DC vaccines in clinical trials are based on MoDCs. In particular, DCV trials in GBM now all use MoDCs. The common method is to collect autologous monocytes from patients, induce them to differentiate into immature DCs *in vitro*, expose them to TAAs after induction of maturation, and then transfuse them into the same patient. In trials that used mDCs, DC maturation was mostly induced by GM-CSF combined with IL-4, PGE2, TNF-α, or IFN-γ (95–99). There are couples of trials that induce DC maturation by using IL-6, IL-1β, TNF-α, or PGE2 without GM-CSF (100–102). Although cDCs may be superior to MoDCs in their ability to stimulate T cells (7, 103), there are currently no established protocols for isolating or differentiating these cells *in vitro*. Nonetheless, in some diseases, most notably melanoma, the use of cDCs and pDCs as DC vaccines has shown some encouraging early results that may be extended to GBM research in the future (104–107).

In addition, since iDCs are less capable of stimulating T cells than mDCs and may even induce tolerance, mDCs are used in most DCV trials. However, there are trials using iDCs that have reported clinical benefits (108, 109).

### 4.2 Tumor-associated antigens

By priming CD8+ T cells against TAA, DCs are an important part in antitumor immunity. Thus, the efficacy of DCV is related to the existence of TAAs, also known as neoantigens, in individual tumors. The overall mutational burden of GBM is very low, but patients who relapse after TMZ chemotherapy have an increased mutational burden (110).

Previous trials using DCV used tumor lysates, tumor cell apoptotic bodies, irradiated tumor cells, DC-tumor cell fusion, and tumor cell surface eluted peptides as whole tumor cell TAAs. Whole tumor cell-derived TAAs contain numerous TAAs, assuring the diversity of antigens and reducing the risk of TAA-loss variants escaping (111). However, due to the immunosuppressive factors produced by glioma cells, whole tumor cell-derived TAAs may inhibit the DC differentiation and maturation or alter the function of generated DCs (112). Furthermore, whole tumor cell-derived TAA vaccines produced using current methods are poorly immunogenic and difficult to induce potent and durable T cell responses (113).

Some DCV studies use molecularly defined TAAs, including specific peptides, proteins, and DCs transfected with TAA-coding mRNA. The source of molecularly defined TAAs is more standardized and reproducible, making it easier to monitor target-specific responses. In addition, they can be personalized for different individuals (114). However, compared to TAAs derived from whole tumor cells, molecularly defined TAAs lack diversity. Therefore, to reduce the risk of escape of TAA-loss mutants, several molecularly defined TAAs should be used.

### 4.3 Dose and route of application

To induce a T cell response in a healthy subject, the minimum DC dose is 2×10^^6^ DC/vaccine (115), while no study to date has achieved dose-limiting toxicity. While several clinical studies aiming at determining the best dose of DCV therapy have been conducted previously, and some of them have been completed (e. g. NCT00612001, NCT01171469, NCT00068510, NCT00107185), the relationship between clinical outcomes and DC dose, and the dose-response relationship of the optimal dose have remained inconclusive. Studies have shown that patients receiving lower doses of DC have longer survival (116); while some studies suggest that improving the efficiency of DC migrating to lymph nodes may increase patient survival (117). This may be because the DCV used in these studies was handled differently as well as the status of DC, making it difficult to compare to derive the optimal dose of DC. In the existing clinical trials, almost all patients received multiple vaccinations, mainly using the prime-boost method (18). Several studies have reported a trend toward improved survival in booster recipients (102).

Different routes of injection of DCV result in different distributions of DC *in vivo (* 118, 119). Currently, the routes of administration used in clinical trials of DCV include intravenous injection, subcutaneous injection, and nodal injection. Subcutaneous injection is by far the most common route of administration, with up to 4% of DCV reaching the draining lymph nodes. Irrespective of the routes of administration, high numbers of DCs remained at the injection site, lost viability, and were eliminated by infiltrating CD163+ macrophages within 48 hours (120). The intranodal injection may allow more DCs to migrate to the T-cell region, but whether it is more effective in inducing antigen-specific immune responses remains to be determined (120).

### 4.4 Treatment options

Most patients underwent cytoreductive surgery before DCV, while some patients underwent biopsy alone or without surgery. The extent of surgical resection is positively associated with survival (121), while minimal residual disease status also favors DCV therapy (121, 122), which may be related to a reduction in local immunosuppression (123). Yet, other studies have shown that the extent of resection is not related to survival in DCV treatment (124). Therefore, in addition to the absolute volume of the residual tumor, other factors such as the composition of the residual tumor may influence the effect of DCV.

DCV treatment is often combined with radiotherapy or chemotherapy, or both. Tumor cell death after chemoradiotherapy releases tumor antigens, then the brain endothelium presents MHC class I antigens to circulating CD8^+^ T cells, which can enhance the tumor-specific effector CTL homing to brain tumors (125). The most widely used chemotherapeutic agent combined with DCV is TMZ, which has been used in all current DCV-controlled trials. TMZ can improve immunoreactivity by reducing Tregs and interfering with their recruitment to tumors (126). Although TMZ often induces lymphopenia, the lymphocyte zone restored after chemotherapy can still induce an antitumor response (117, 127). The specific efficacy is related to the dose of TMZ: for example, lower doses of TMZ help deplete Tregs, whereas myelosuppressive doses enhance the response to peptide vaccines (128). However, there is also evidence that CD8^+^ T cells expanded by DCV previously may be depleted by TMZ (100, 129). Moreover, only in the absence of TMZ was DCV able to generate IFN-γ-producing effector memory T cells, which was positively related to survival (130). Thus, the effect of TMZ on DCV efficacy remains inconclusive.

### 4.5 Safety

By far, no serious vaccine-related adverse events have been observed, except for a few studies that reported severe adverse events (grade ≥3) according to the National Cancer Institute Common Toxicity Criteria (NCI CTC). Some of these adverse events were severe peritumoral edema leading to other neurological symptoms (96, 131); some were allergies following co-injection of DCV and GM-CSF (132).

Commonly observed adverse reactions attributed to DCV are generally mild (≤ grade 2), including induration, pain, pruritus, and erythema in injection sites, as well as meningeal irritation, lymph node swelling, flu-like symptoms, edema, etc (95, 97, 98, 101, 102, 116, 133–143). These symptoms may be caused by disease progression or other concomitant therapies as well. All in all, DCV therapy was well tolerated as a therapeutic method (for a brief introduction of DCV clinical trials registered in clinical trials.gov, see **Table 2** and **Table 3**).

**Table 2:** Completed clinical trials registered on clinicaltrials.gov concerning dendritic cell vaccine in glioma patients.

Clinical trial	Strategy	Condition	Phase	Combinatorial treatment
NCT00576446	Autologous DCs loaded with tumor lysate	Malignant glioma	I	Gliadel Wafer
NCT01792505	Autologous DCs loaded with tumor lysate	Malignant glioma	I	Imiquimod
NCT01808820	Autologous DCs loaded with tumor lysate	Malignant glioma	I	Imiquimod
NCT00766753	Autologous DCs loaded with TAA or TAA-derived peptides	Recurrent malignant glioma	I/II	Poly-ICLC
NCT00576641	Autologous DCs loaded with TAA or TAA-derived peptides	Brain stem glioma and glioblastoma	I	N/A
NCT00576537	Autologous DCs loaded with tumor lysate	Glioblastoma	II	N/A
NCT01213407	Autologous DCs loaded with tumor lysate	Glioblastoma	II	Standard therapy
NCT00612001	Autologous DCs loaded with TAA or TAA-derived peptides	Malignant glioma	I	N/A
NCT01171469	Autologous DCs loaded with TAA or TAA-derived peptides	Malignant glioma	I	Imiquimod
NCT02010606	Autologous DCs loaded with tumor lysate	Glioblastoma	I	Temozolomide, radiotherapy, bevacizumab
NCT00068510	Autologous DCs loaded with tumor lysate	Malignant glioma	I	N/A
NCT01006044	Autologous DCs loaded with tumor lysate	Glioblastoma	II	Standard therapy
NCT02709616	Autologous DCs pulsed with TAA-coding RNAs	Glioblastoma	I	Temozolomide, concurrent radiotherapy
NCT00323115	Autologous DCs	Glioblastoma	II	Temozolomide, radiotherapy
NCT01635283	Autologous DCs loaded with tumor lysate	Low-grade glioma	II	N/A
NCT00107185	Autologous DCs loaded with tumor lysate	Malignant glioma	I	N/A
NCT01291420	Autologous DCs pulsed with TAA-coding RNAs	Glioblastoma	I/II	N/A
NCT02049489	Autologous DCs loaded with TAA or TAA-derived peptides	Recurrent glioblastoma	I	N/A
NCT03615404	Autologous DCs pulsed with TAA-coding RNAs	Malignant glioma	I	Temozolomide, standard radiotherapy, GM-CSF
NCT02820584	Autologous DCs loaded with GSCs	Glioblastoma	I	N/A
NCT00846456	Autologous DCs pulsed with TAA-coding RNAs	Glioblastoma	I/II	Standard therapy
NCT03360708	Autologous DCs pulsed with tumor lysate	Recurrent glioblastoma	I	N/A
NCT00626483	Autologous DCs pulsed with TAA-coding RNAs	Glioblastoma	I	Basiliximab, GM-CSF
NCT02366728	Autologous DCs pulsed with TAA-coding RNAs	Newly-diagnosed glioblastoma	II	Basiliximab, tetanus-diphtheria toxoid
NCT00890032	Autologous DCs pulsed with TAA-coding RNAs	Recurrent glioblastoma	I	N/A
NCT01280552	Autologous DCs loaded with tumor-derived peptides	Glioblastoma	II	Chemotherapy
NCT00639639	Autologous DCs pulsed with TAA-coding RNAs	Newly-diagnosed glioblastoma	I	Tetanus toxoid
NCT01522820	DC-protein fusion	Glioma	I	Rapamycin
NCT00693095	Autologous DCs pulsed with TAA-coding RNAs	Glioblastoma	I	Temozolomide, radiotherapy

N/A, Not available.

**Table 3:** Ongoing clinical trials registered on clinicaltrials.gov concerning dendritic cell vaccine in glioma patients.

Clinical trial	Status	Strategy	Condition	Phase	Combinatorial treatment
NCT04911621	Not recruiting	Autologous DCs pulsed with TAA-coding RNAs	Pediatric high-grade glioma, diffuse intrinsic pontine glioma	I/II	Temozolomide, conventional next-line treatment
NCT03334305	Not recruiting	Autologous DCs pulsed with TAA-coding RNAs	Pediatric high-grade glioma	I	Temozolomide, GM-CSF
NCT04837547	Recruiting	Autologous DCs pulsed with TAA-coding RNAs	Newly-diagnosed diffuse intrinsic pontine glioma	I	Autologous T cells
NCT01204684	Not recruiting	Autologous DCs loaded with tumor lysate	Glioma	II	resiquimod, poly-ICLC
NCT04552886	Recruiting	Autologous DCs	Glioblastoma	I	N/A
NCT04388033	Recruiting	DC-cancer cell fusion	Glioblastoma	I/II	IL-12, temozolomide
NCT00045968	Not recruiting	Autologous DCs loaded with tumor lysate	Glioblastoma	III	N/A
NCT04523688	Recruiting	Autologous DCs loaded with tumor lysate	Glioblastoma	II	Temozolomide
NCT02649582	Recruiting	Autologous DCs pulsed with TAA-coding RNAs	Glioblastoma	I/II	Temozolomide
NCT05457959	Not yet recruiting	Autologous DCs loaded with TAA or TAA-derived peptides	Recurrent and/​or progressive diffuse hemispheric glioma	I	Nivolumab, Ipilimumab
NCT03879512	Recruiting	Autologous DCs loaded with tumor lysate	Recurrent pediatric high-grade glioma	I/II	Metronomic cyclophosphamide, nivolumab/Ipilimumab
NCT03548571	Not recruiting	Autologous DCs pulsed with TAA-coding RNAs	Glioblastoma	II/III	Temozolomide
NCT04801147	Recruiting	Autologous DCs loaded with tumor lysate	Glioblastoma	I/II	Temozolomide, radiotherapy
NCT04115761	Recruiting	Autologous DCs loaded with TAA or TAA-derived peptides	Glioblastoma	II	Radiotherapy, chemotherapy
NCT03395587	Recruiting	Autologous DCs loaded with tumor lysate	Newly-diagnosed glioblastoma	II	Standard therapy
NCT03396575	Recruiting	Autologous DCs pulsed with TAA-coding RNAs	Brain stem glioma	I	Radiotherapy, temozolomide, GM-CSF
NCT02465268	Not recruiting	Autologous DCs pulsed with TAA-coding RNAs	Newly-diagnosed glioblastoma	II	Tetanus-diphtheria toxoid, GM-CSF
NCT03400917	Not recruiting	Autologous DCs loaded with TAA or TAA-derived peptides	Newly-diagnosed glioblastoma	II	GM-CSF, chemoradiotherapy
NCT04963413	Recruiting	Autologous DCs pulsed with TAA-coding RNAs	Newly-diagnosed glioblastoma	I	GM-CSF
NCT01957956	Not recruiting	Autologous DCs loaded with tumor lysate	Glioblastoma	I	Temozolomide
NCT04201873	Recruiting	Autologous DCs loaded with tumor lysate	Recurrent glioblastoma	I	Pembrolizumab, poly-ICLC
NCT05100641	Not yet recruiting	Autologous DCs loaded with TAA or TAA-derived peptides	Glioblastoma	III	Temozolomide, radiotherapy
NCT04968366	Recruiting	Autologous DCs loaded with TAA or TAA-derived peptides	Newly-diagnosed glioblastoma	I	Temozolomide
NCT03688178	Recruiting	Autologous DCs pulsed with TAA-coding RNAs	Glioblastoma	II	Temozolomide, varlilumab
NCT04888611	Recruiting	Autologous DCs loaded with TAA or TAA-derived peptides	Recurrent glioblastoma	II	Carilizumab

N/A, Not available.

## 5 Optimizing DCV therapy

### 5.1 Develop Other DC-derived vaccines

Although most DCVs use MoDCs that have been induced to differentiate *in vitro*, long-term *in vitro* culture can result in decreased MoDC migration and functional loss (7). Therefore, MoDC is probably not the best DC subpopulation for vaccine manufacturing, and the development of vaccines based on naturally circulating DC subtypes such as cDC, pDC, or Langhans cell may achieve better results. Among these DC subsets, it has been proven that cDCs have a stronger ability to induce CD8^+^ T cell response (7, 103). To date, the difficulty of producing cDC1/2 in large quantities from patients remains an obstacle to cDC-based DCVs. Therefore, future efforts should focus on solving the technical and cost issues of generating large numbers of cDCs.

### 5.2 Screen specific immune biomarkers

ICIs have achieved clinical success in effectively treating various cancers, which are related to specific immune biomarkers to guide application. Immune biomarkers such as tumor mutation burden and PD-L1 positivity provide accurate and non-invasive means for patient preselection (13, 15, 144), which are of great value to the success of antitumor immunotherapy. Unfortunately, the lack of strong patient-preselected biomarkers immensely limits the guide of application of DCV; therefore, there is a surge in urgency to screen out biomarkers that are most likely to predict a positive patient response to DCV. The selection of patient subgroups by specific biomarkers that improve the likelihood of a subject’s response to DCV will help guide the design of clinical trials.

### 5.3 Improve the function of DC in the glioma microenvironment

The glioma microenvironment is composed of various immunosuppressive cells, all of which are of great importance in disease progression. Targeting only one type of cell is not sufficient to modify the entire TME. Therefore, to improve DC function in the TME, it may be necessary to combine it with a variety of other immunotherapy methods to get over the negative effects of immunosuppression and immune checkpoint modulation.

In preclinical models, anti-CD25 antibodies are commonly used to deplete Tregs (145–147) or limit their immunosuppressive function by blocking molecules like PD-1, CTLA-4, and Tim-3 or enzymes such as IDO (146, 148, 149). However, it has been reported in murine models that high-dose unfractionated radiotherapy or low-dose TMZ or cyclophosphamide chemotherapy can deplete Tregs (150, 151). An encouraging approach is reducing the effect of Tregs by combining radiotherapy with anti-IDO, which eventually improves the survival of mice (152). When anti-CD25 therapy is combined, beneficial effects on survival caused by DCV have been reported by several other studies, especially when Tregs are depleted before vaccination (145, 153).

*In vitro*, paclitaxel promoted MDSC differentiation into DCs in a TLR4-independent manner (154). Docetaxel induces the transformation of MDSCs into M1-like macrophages and selectively enhances CTL responses (155). All-trans retinoic acid can promote MDSC maturation (156). In addition, low doses of 5-FU (157), capecitabine (158), etc. can deplete MDSCs. Pexidatinib reduced MDSCs and M2-like TAMs by blocking CSF-1 receptor signaling (159), while STAT3 inhibitors reduced MDSCs and impaired their function (160).

Blocking the CSF-1/CSF-1R axis prevents monocyte differentiation, thereby reducing the number of TAMs, while also reducing the survival of existing TAMs (161, 162). Blockade of the CCL2/CCR2 axis inhibited monocyte recruitment but did not affect the TAMs formed (163, 164). CD47 is a “don’t eat me” signal, and blocking CD47/SIRP enhances TAM phagocytosis of tumor cells (165). Oncolytic virotherapy repolarizes M2-like TAMs to M1-like TAMs (166, 167).

Although ICIs have achieved impressive results in various tumors, and immune checkpoint inhibitors have improved survival in a mouse GBM model (146, 148, 149, 168), ICIs alone have not been effective in the treatment of GBM (17, 169). However, the combination of ICI and DCV was more effective than DCV alone. Currently, the most commonly used target of ICIs is PD-1, followed by CTLA-4 (10).

### 5.4 New routes of administration

To date, there haven’t been any reported clinical trials for glioma using intratumoral injection of DCV yet. It has been shown that in an orthotopic GL261 glioma murine model, compared with subcutaneous injection of GL261 lysate-loaded DCs, intratumoral injection is less effective; however, combining these two administration routes is more effective than subcutaneous injection alone (170). Intratumoral injected DCs could be detected in the tumor parenchyma while not in the cervical lymph nodes. Therefore, intratumoral injection of DC may have a distinct mechanism to improve survival. This may be because intratumoral DC injection enhances the anti-tumor immune response induced by subcutaneous injection of DC by pro-immunomodulating cytokines in the TME, reducing Treg cells, and directly inhibiting tumor proliferation by TNF (170, 171). Therefore, combining the two in clinical trials may lead to better results.

## 6 Conclusion

Thousands of glioma patients have been treated with DCV over the past few decades. During this period of time, the methods of production and treatment of DCV have also been gradually diversified. Due to the weak immunogenicity of DCV produced by conventional methods, which cannot induce strong and durable T-cell responses, many efforts have been made to improve their immunogenicity (172, 173). Yet, those DCVs with higher immunogenicity don’t seem to be as clinically successful as expected. Thus, whether there is a better way to improve immunogenicity or whether immunogenicity doesn’t take a crucial part in the effect of DCV remains a question. Although no definitive conclusion can be made about the efficacy of DCV, some promising results still show the great potential of DCV as a therapeutic tool for GBM. To conclude, the reasons why the clinical application of DCV is not as good as expected may be related to the limitation of DC function by the immunosuppressive microenvironment, the lack of optimal dosage standards, and the lack of specific immune biomarkers. Either way, if future studies address the above issues, DCV will have a significant impact on GBM treatment and significantly improve patient outcomes.

## Author contributions

SZ: Writing – review & editing. YZ: Writing – original draft. XM: Writing – review & editing. SF: Writing – review & editing. HZ: Writing – review & editing. XC: Writing – review & editing. XY: Writing – review & editing. KS: Writing – review & editing.
